# Abnormal Protein Glycosylation and Activated PI3K/Akt/mTOR Pathway: Role in Bladder Cancer Prognosis and Targeted Therapeutics

**DOI:** 10.1371/journal.pone.0141253

**Published:** 2015-11-16

**Authors:** Céu Costa, Sofia Pereira, Luís Lima, Andreia Peixoto, Elisabete Fernandes, Diogo Neves, Manuel Neves, Cristiana Gaiteiro, Ana Tavares, Rui M. Gil da Costa, Ricardo Cruz, Teresina Amaro, Paula A. Oliveira, José Alexandre Ferreira, Lúcio L. Santos

**Affiliations:** 1 Experimental Pathology and Therapeutics Group, Portuguese Institute of Oncology, Rua Dr. António Bernardino de Almeida, Porto, Portugal; 2 ICBAS, Abel Salazar Biomedical Sciences Institute, University of Porto, Porto, Portugal; 3 Health Sciences Faculty of University Fernando Pessoa, Porto, Portugal; 4 Nucleo de Investigação e Informação em Farmácia - Centro de Investigação em Saúde e Ambiente (CISA), School of Allied Health Sciences – Polytechnic Institute of Oporto, Porto, Portugal; 5 Institute of Pathology and Molecular Immunology of the University of Porto (IPATIMUP), Porto, Portugal; 6 Department of Pathology, Portuguese Institute of Oncology, Porto, Portugal; 7 Faculty of Engineering, Laboratory for Process, Environment, Biotechnology and Energy Engineering (LEPABE), University of Porto, Porto, Portugal; 8 Department of Urology, Portuguese Institute of Oncology, Porto, Portugal; 9 Department of Urology, Hospital Pedro Hispano, Matosinhos, Portugal; 10 Department of Veterinary Sciences, CITAB, University of Trás-os-Montes and Alto Douro, Vila Real, Portugal; 11 Mass Spectrometry Center of the University of Aveiro, Campus de Santiago, Aveiro, Portugal; 12 Department of Surgical Oncology, Portuguese Institute of Oncology, Porto, Portugal; Centro Nacional de Investigaciones Oncológicas (CNIO), SPAIN

## Abstract

Muscle invasive bladder cancer (MIBC, stage ≥T2) is generally associated with poor prognosis, constituting the second most common cause of death among genitourinary tumours. Due to high molecular heterogeneity significant variations in the natural history and disease outcome have been observed. This has also delayed the introduction of personalized therapeutics, making advanced stage bladder cancer almost an orphan disease in terms of treatment. Altered protein glycosylation translated by the expression of the sialyl-Tn antigen (STn) and its precursor Tn as well as the activation of the PI3K/Akt/mTOR pathway are cancer-associated events that may hold potential for patient stratification and guided therapy. Therefore, a retrospective design, 96 bladder tumours of different stages (Ta, T1-T4) was screened for STn and phosphorylated forms of Akt (pAkt), mTOR (pmTOR), S6 (pS6) and PTEN, related with the activation of the PI3K/Akt/mTOR pathway. In our series the expression of Tn was residual and was not linked to stage or outcome, while STn was statically higher in MIBC when compared to non-muscle invasive tumours (p = 0.001) and associated decreased cancer-specific survival (log rank p = 0.024). Conversely, PI3K/Akt/mTOR pathway intermediates showed an equal distribution between non-muscle invasive bladder cancer (NMIBC) and MIBC and did not associate with cancer-specif survival (CSS) in any of these groups. However, the overexpression of pAKT, pmTOR and/or pS6 allowed discriminating STn-positive advanced stage bladder tumours facing worst CSS (p = 0.027). Furthermore, multivariate Cox regression analysis revealed that overexpression of PI3K/Akt/mTOR pathway proteins in STn+ MIBC was independently associated with approximately 6-fold risk of death by cancer (p = 0.039). Mice bearing advanced stage chemically-induced bladder tumours mimicking the histological and molecular nature of human tumours were then administrated with mTOR-pathway inhibitor sirolimus (rapamycin). This decreased the number of invasive lesions and, concomitantly, the expression of STn and also pS6, the downstream effector of the PI3K/Akt/mTOR pathway. In conclusion, STn was found to be marker of poor prognosis in bladder cancer and, in combination with PI3K/Akt/mTOR pathway evaluation, holds potential to improve the stratification of stage disease. Animal experiments suggest that mTOR pathway inhibition could be a potential therapeutic approach for this specific subtype of MIBC.

## Introduction

Bladder cancer is the second most deadly genitourinary tumour and presents significantly worse prognosis upon *muscularis propria* invasion [1]. Approximately 20–30% of the newly diagnosed cases are muscle invasive bladder cancers (MIBC; T2-T4 stages), while 50% are non-muscle invasive bladder tumours (NMIBC) with high potential to progress to invasion. MIBC treatment includes cystectomy and (neo)adjuvant cisplatin-based chemotherapy regimens [2]. However, significant variations in the natural history of the disease and responses to treatment can be observed between tumours with identical histological features, reflecting their high molecular heterogeneity [3]. Furthermore, approximately 50% of cases develop metastasis within 5 years, urging the identification of biomarkers to assist prognostication and the development of more effective targeted therapeutics [4].

**Fig 1:**
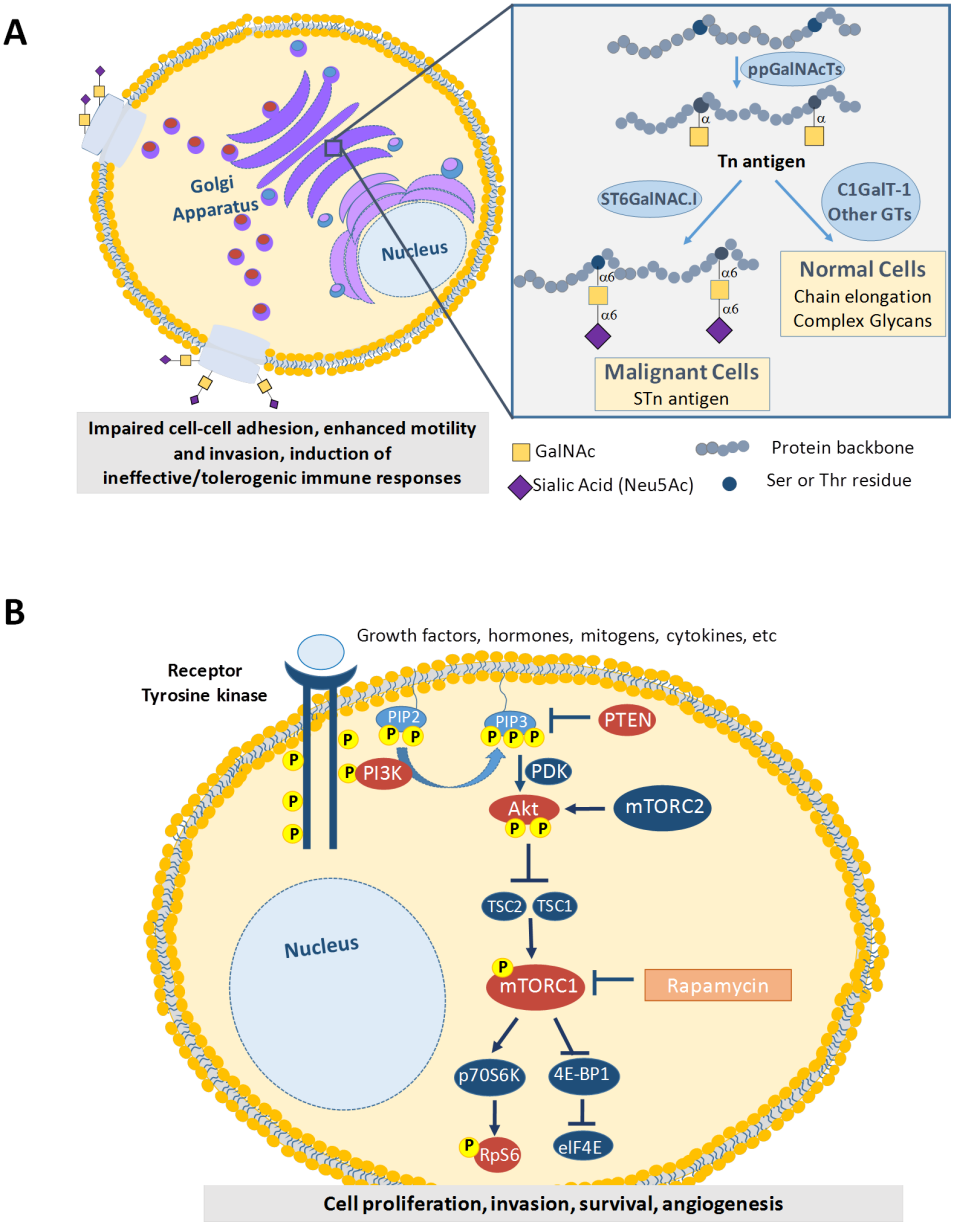
Schematic representation of membrane protein *O*-glycosylation and the PI3K/Akt/mTOR pathways. A) Representation of membrane protein *O*-glycosylation with emphasis on the STn expression by cancer cells. This is a highly regulated process of critical importance for protein stability and function. Briefly, newly synthesized proteins are *O*-glycosylated in the Golgi apparatus by the ppGalNAcTs-mediated addition of GalNAc moiety to Ser/Thr residues. This originates the Tn antigen (GalNAc-O-Ser/Thr-protein backbone), which is the simplest O-glycan. In normal cells these chains are extended through the sequential addition of other sugars first by CGALT-1 and then other enzymes. This culminates in highly complex, heterogeneous and elongated glycans often terminated by ABO or Lewis blood group related antigens (left drawing). In cancer cells the Tn antigen is immediately sialylated by ST6GalNAc.I, originating the STn antigen (Neu5Ac-GalNAc-O-Ser/Thr-protein backbone), thereby inhibiting further chain elongation (right drawing). The expression of STn at the cell surface influences cell-cell adhesion and cancer cell recognition, favouring motility, invasion and immune escape. B) Schematic representation of the PI3K/Akt/mTOR pathway, which is ubiquitously activated in bladder tumours. This is a highly conserved pathway regulated mainly by a wide variety of extracellular signals, including mitogenic growth factors, hormones, nutrients, cellular energy levels, and stress conditions. These signals activate tyrosine receptor kinases that recruit PI3K, which catalyses the conversion of membrane-bound PIP2 to PIP3. Then Akt and PDK-1 are activated through binding to PIP3. PTEN preferentially dephosphorylates PIP3, inhibiting signalling progression. Full Akt activation requires double phosphorylation by PDK-1 itself and PDK-2 (not shown). Akt phosphorylates mTOR directly or may also inactivate TSC1/TSC2 complex, inhibiting mTOR inactivation. mTORC1 triggers cell growth and proliferation by phosphorylating eukaryotic translation regulators, among these p70S6 kinase (p70S6K or S6K1) that, in turn, phosphorylates the ribosomal protein S6 (pS6), and the eukaryotic translation initiation factor 4E–binding protein 1 (4E-BP1). For the protein mTOR to activate its signalling cascade, it must form the rapamycin-sensitive ternary complex mTORC1. Key PI3K/Akt/mTOR-pathway proteins pAkt, pmTOR and pS6 explored in this studied are highlighted by orange circles.

To meet this need, we have recently addressed the expression of the cancer-associated sialyl-Tn antigen (STn) on a small prospective series of unselected bladder cancer patients [5]. STn is an abnormal post-translational modification that results from a premature stop in cell-membrane proteins *O*-glycosylation by sialylation of the Tn antigen (Fig 1A). In bladder tumours, STn it was mainly present in advanced stage cases, while absent from most low-grade NMIBC [5]. Moreover, it was not expressed by the normal urothelium, denoting a cancer-specific nature [5]. Studies *in vitro* showed that STn expression endowed bladder cancer cells with high invasion capability [5] and an immunotolerogenic phenotype, potentially favoring disease dissemination [6]. Alterations in cell-surface protein glycosylation have be implicated in the activation of intracellular oncogenic signalling pathways [7], including the phosphoinositide-3 kinase (PI3K)/Akt signalling pathway [8] which is thought to play a critical role in bladder cancer development. These preliminary observations support the hypothesis that STn expression may play a key role in disease outcome, which warrants a deeper investigation. Several studies also suggest that Tn antigen, which is a precursor of STn, may be also implicated in oncogenic events [7]; however nothing is known about the expression of this glycan in bladder tumours.

The phosphatidylinositol-3-kinase (PI3K)/Akt and the mammalian target of rapamycin (mTOR) pathways are interconnected signaling cascades essential for bladder cell growth and survival (Fig 1B). The PI3K/Akt/mTOR or mTOR pathway integrates a multiplicity of extracellular signals to regulate downstream signaling and protein synthesis, which ultimately leads to a competitive growth advantage, metastatic competence, angiogenesis, and therapy resistance [9]. The signaling cascade begins with PI3K activation in the cell membrane followed by serine/threonine kinase Akt cell membrane translocation and activation. The best studied downstream substrate of Akt is the serine/threonine kinase mTOR, whose downstream effector is S6 kinase-1 (S6K1). In particular, a subset of mTOR pathway alterations have been shown to occur in bladder cancer, such as mutations in *PIK3CA* gene, which culminates with increased mTOR signaling and bladder cancer cells resistance to apoptosis [10]. Moreover, the pharmacological or biochemical inhibition of the PI3K pathway drastically reduced the invasive capacity of bladder cancer cell lines. Furthermore, over half of primary human bladder tumours present high Akt phosphorylation and the aberrant activation of this pathway has been suggested to contribute to invasion [11]. Another event influencing mTOR pathway activation in bladder tumours involves the loss of tumor suppressor PTEN (phosphatase and tensin homolog deleted on chromosome ten) function [12]. PTEN normally suppresses activation of the PI3K/Akt/mTOR pathway antagonizing PI3K and preventing activation of Akt and PDK-1. PTEN also functions to regulate chemotaxis and cell motility, thereby promoting tumor invasion [13]. In summary, there are evidences that a comprehensive evaluation of PI3K/Akt/mTOR pathway associated proteins may hold significant potential for value for patient stratification. Moreover, many preclinical and clinical studies support that mTOR inhibitors, such as sirolimus (rapamycin) and their derivatives may improve cancer treatment [13,14].

Based on these observations we hypothesize that Tn and/or STn may act synergistically with the mTOR pathway to drive bladder cancer progression. As such, we have devoted to evaluating the expression of STn and proteins associated with the activation of the PI3K/Akt/mTOR pathway activation in bladder tumours at different stages. We anticipate that the combination of extracellular and intracellular oncogenic events may improve patient stratification and provide insights for novel therapeutics. Furthermore we have estimated the impact of sirolimus in chemically-induced urothelial tumours in mice, envisaging the creation of a rationale for more effective bladder cancer therapeutics.

## Materials and Methods

### Ethics Statement

This work involves experiences in tumour samples of patients diagnosed with bladder cancer in the Portuguese Insitute of Oncology of Porto. All procedures were performed after patient’s written informed consent and approved by the Ethics Committee of Portuguese Institute of Oncology—Porto. All clinicopathological information was obtained from patients’ clinical records.

It also involves animal experiments. All procedures involving animals were performed in accordance with the European Directive 2010/63/EU. During the course of this study, the animals were fed *ad libitum* with standardized food (Tecklad Global Diet, Harlan, Spain). The following protocol was approved by the Portuguese Ethics Committee for Animal Experimentation (Direção Geral de Veterinária, Approval no. 520/000/000/2003). All mice used in the experiment were acclimatized for one week under routine laboratory conditions before starting the experiments. They were housed randomly in groups of 4–5 in plastic cages, with hard wood chips for bedding. The animals were maintained in a room with a controlled temperature of 23±2°C, a 12-hour light/dark cycle and 55±5% humidity. The animals' drinking solutions were changed once a week or earlier if necessary, and the volume drunk was recorded. Weekly food intake was also noted. All mice were monitored throughout the experiment for signs of distress and loss of body weight. The animals were sacrificed with 0.4% sodium pentobarbital (1 ml/Kg, intraperitoneal).

### Population

This study was performed in a retrospective series of 96 formalin-fixed paraffin-embedded bladder tumours obtained from archived paraffin blocks at the Portuguese Institute of Oncology—Porto (IPOP), Portugal. Bladder tumours were extracted from 82 men and 14 women, ranging in age from 38 to 92 years (median of 69.5 years), admitted and treated at the IPOP between 2005 and 2007. Forty seven of the examined tumours were histologically classified as NMBIC (Ta and T1) and 49 as invasive lesions (T2-T4). Sixteen were low grade and 80 were high grade tumours, according to the 2004 WHO grading criteria. Furthermore, carcinoma *in situ* (CIS) was found concomitantly in 20.8% of the patients. The average follow up time period was 45 months (1–134 months). Cystectomy was performed in 64 patients (66.7%) while the other 32 (33.3%) were submitted to transurethral resection. Lymphadenectomy was performed in approximately 47% of the patients and from those 37% presented metastasis. Fifty four (56.3%) tumours were primary and 42 (43.7%) were recurrent tumors. From the recurrent tumours, 38% had no prior treatment, 27% were treated with Mitomicin C, 11% with BCG and 19% were submitted to both treatments. Moreover 5% of these patients were treated with neoadjuvant chemotherapy prior to the cystectomy. Table 1 summarizes the clinicopathological information.

**Table 1:** Clinical-pathological data of the studied sample (*n* = 96).

**Age**, *years*		
	median [min—max]	69.5 [38–92]
**Gender**, *n* (%)		
	Male	82 (85.4%)
	Female	14 (14.6%)
**Stage**, *n* (%)		
	Ta	27 (28.1%)
	T1	20 (20.8%)
	T2	9 (9.4%)
	T3	20 (20.8%)
	T4	20 (20.8%)
**Grade, n (%)**		
	**Low**	16 (16.7%)
	**High**	80 (83.3%)
**Recurrence status, n(%)**		
	**Primary**	54 (56.3%)
	**Recurrent**	42 (43.7%)
**Associated Cis, n(%)**		
	**No**	76 (79.2%)
	**Yes**	20 (20.8%)
**Metastasis, n(%)**		
	**No**	19 (63.3%)
	**Yes**	11 (36.7%)
**Follow-up,** *n (%)*		
	Alive, lost or death from other causes	67 (69.8%)
	Death from cancer	29 (30.2%)

Cancer-specific survival (CSS) was defined as the period between the tumour removal by surgery and either patient death by cancer or the last follow-up information. All procedures were performed after patient’s informed consent and approved by the Ethics Committee of IPO-Porto. All clinicopathological information was obtained from patients’ clinical records.

### Immunohistochemistry

The expressions of STn antigen, its precursor Tn, and phosphorylated forms of Akt (pAkt), mTOR (pmTOR), S6 (pS6) and PTEN in bladder tumours were accessed by immunohistochemistry using the avidin/streptavidin peroxidase method, as described by Ferreira et al. [5]. Information on the primary antibodies and dilutions used in this study are summarized in Table 2. Immunoreactivity was revealed using diaminobenzidine (DAB, Thermo Scientific LabVision) as chromogen and sections were counterstained with Harris’s hematoxylin. Negative controls were performed by replacing the primary antibody with 5% bovine serum albumin (BSA). Positive controls were known positive tissues for the antigens under study.

**Table 2:** Antibodies used in the immunohistochemical analysis.

Antibody	Vendor	Clone	Dilution
Tn	Non-commercial Hybridoma*	IE3	1:5
STn	Non-commercial Hybridoma*	TKH2	1:20
Ki-67	Dako	MIB-1	1:100
p53	Dako	DO-7	1:100
Phos-AKT	Cell Signaling	Ser473 (736E11)	1:50
Phos-mTOR	Cell Signaling	Ser2448(49F9)	1:100
Phos-S6	Cell Signaling	Ser240/244 polyclonal	1:75
PTEN	Cell Signaling	D4.3 XP	1:50

*Kindly provided by Prof. Celso Reis (IPATIMUP, UP, Portugal)

### Immunohistochemistry scoring of human tumours

The immunostained sections were assessed double-blindly by light microscopy by two independent observers (CC and SP) and validated by an experienced pathologist (TA). Disaccording readings were re-analyzed using a double-headed microscope (Olympus BX46; Olympus Corporation), and consensus was reached. A semi-quantitative approach was established to score the immunohistochemical labeling based on the extent and intensity of the staining.

Given the absence of Tn and STn in the healthy urothelium [5], tumours were classified as positive for these antigens when membrane and/or cytoplasmic immunoreactivity were observed in more than 5% of the tumour, as described by Ferreira et al. [5,15]. pAkt, pmTOR, pS6 and PTEN expressions were scored according to the staining intensity (weak-1 point; moderate-2 points; strong-3 points) multiplied by the percentage of positive cells (0–5%-0 points; >5–25%-1 point; >25–50%-2 points; >50–75%-3 points; >75–100%-4 points). Based on the classification proposed by Nishikawa et al. [16], tumours with a score <6 were considered negative, whereas those with a score≥6 were classified as positive (overexpression). pAkt was evaluated based on nuclear immunoreactivity, pmTOR and pS6 based on cytoplasmic expression and PTEN on both cytoplasmic and nuclear staining, as suggested by other publications [17,18].

### Animal experiments with sirolimus and immunohistochemistry scoring

Histological sections of Imprinting Control Region (ICR) mice bearing N-butyl-N-(4-hydroxybutyl) nitrosamine (BBN)-induced bladder lesions, resulting from our previous work on the impact of sirolimus on bladder cancer [19], were elected for this study. Briefly, four-week-old male ICR mice (25g; Harlan, Barcelona, Spain) were randomly distributed into four groups, as described in detail in a previous publication [18]. Group 1 (n = 6) included mice exposed to 0.05% BBN for 12 weeks followed by tap water for 8 weeks (total of 20 weeks). Group 2 (n = 7) included mice treated with 0.05% BBN solution for twelve weeks, maintained with normal tap water for another week, administrated intraperitoneally with mTOR-inhibitor sirolimus (1.5 mg/kg; Wyeth) for five days a week for six consecutively weeks, i.e. until the 19^th^ week, followed by another week of tap water (total of 20 weeks). Group 3 (n = 6) included mice exposed to 0.05% BBN for 12 weeks followed by tap water for 11 weeks (total of 23 weeks). Group 4 (n = 7) included mice treated with 0.05% BBN and sirolimus, as described for Group 2, but with an exposure to tap water afterwards of 3 weeks (total of 23 weeks). Group 3 and 4 were created to estimate the possibility of late relapse and/or molecular alterations resulting from prolonged survival. All procedures were performed in accordance with the European Directive 2010/63/EU. During the course of this study, the animals were fed *ad libitum* with standardized food (Tecklad Global Diet, Harlan, Spain). The histological changes induced by these experiments included both preneoplastic and neoplastic lesions with invasive potential and invasive tumours, as described in detail by Oliveira et al. [18]. Herein, lesions of high invasive potential and muscle invasive tumours were screened for STn and pS6 by immunohistochemistry, as described in detail for human tumours, since the antibodies used are reactive against both human and mice. Both the intensity and the extension of immunostaining were taken into consideration to score the expression of the antigens, as described in the previous section. The bladder lesions and immunostaining were assessed double-blindly by two independent observers (CC and SP) and validated by an experienced veterinary pathologist (RMGC).

### Statistical analysis

Statistical data analysis was performed with IBM Statistical Package for Social Sciences—SPSS for Windows (version 20.0). Chi-square analysis was used to compare categorical variables. Kaplan-Meier survival curves were used to evaluate correlation between STn expression and cancer-specific survival (CSS) and were compared using log-rank test. Furthermore, multivariate Cox regression analysis was performed to assess the individual effect of the evaluated markers on patient’s survival and adjust to potential confounders (variables that could affect CSS of NMIBC and MIBC patients). The correlation between PI3K/Akt/mTOR pathway molecules was performed using Spearman rho test.

## Results

Altered protein glycosylation, translated by the expression of the STn antigen and its precursor Tn, PI3K/Akt/mTOR pathway molecules (pAkt, pmTOR, pS6), and PTEN inactivation, are salient features of bladder tumours. Herein we have devoted to a comprehensive analysis of these molecular alterations in a series of bladder cancer patients at different stages of the disease, envisaging biomarkers of poor cancer-specific survival.

**Fig 2:**
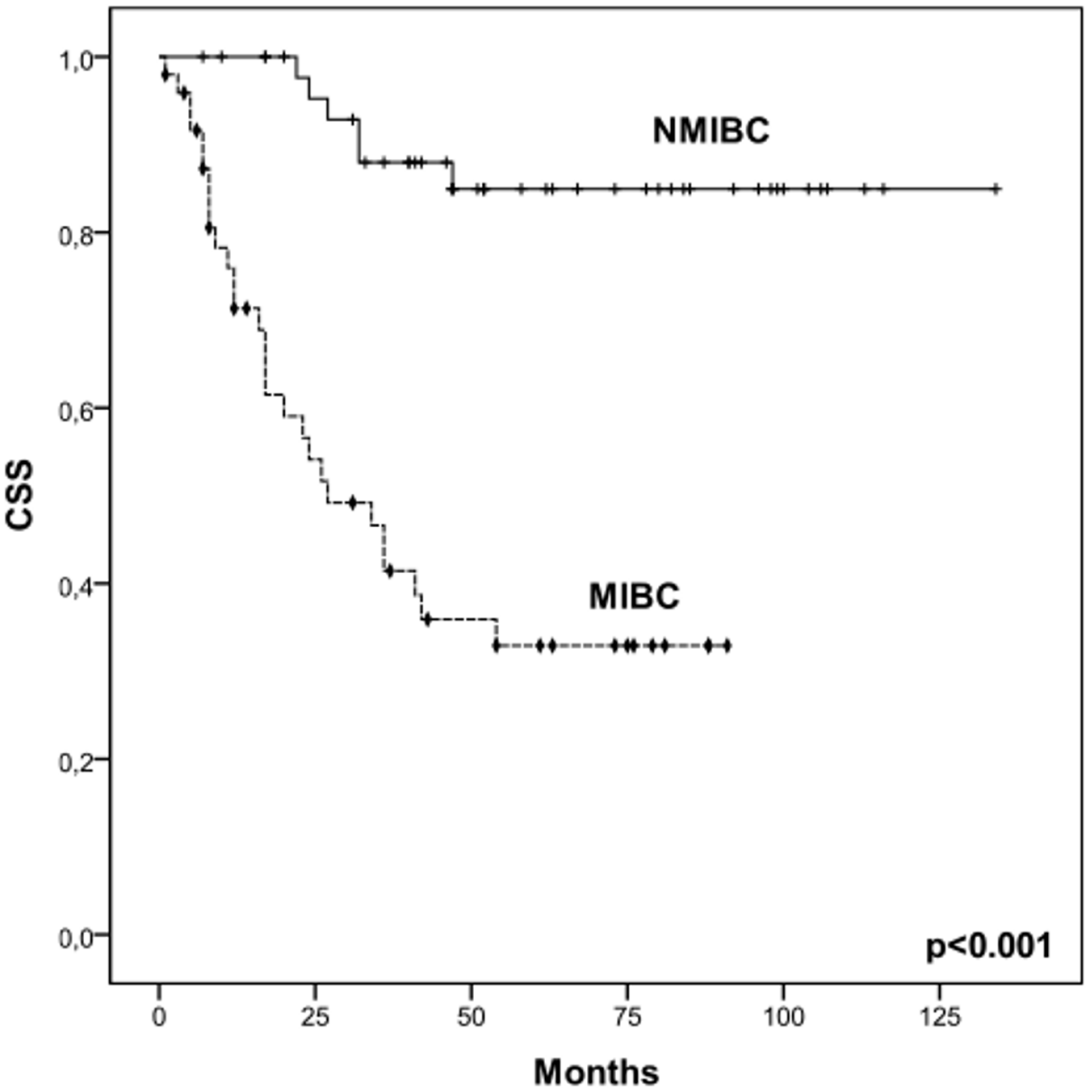
Association between disease groups and cancer-specific survival (CSS) in the studied patients. Kaplan-Meier analysis showing the CSS of NMIBC (Ta and T1) and s of MIBC (T2,T3 and T4). Comparison performed by log-rank test (p<0.001); + censored NMIBC patients; ⧫ censored MIBC patients.

Our dataset was composed by 47 NMIBC and 49 MIBC patients, as showed in Table 1. According to Fig 2, NMIBC presented a higher cancer-specific survival (CSS; mean CSS: 119 months) than MIBC patients (mean CSS: 43 months; log rank, p<0.001). These results demonstrated that our series reflected the natural course of disease and highlighted the significantly lower CSS of MIBC compared to NMIBC cases. Therefore, particular interest was set in the identification of biomarkers for late stage disease based on the comparison between NMIBC and MIBC.

### Tn and STn antigen expressions in bladder cancer

**Fig 3:**
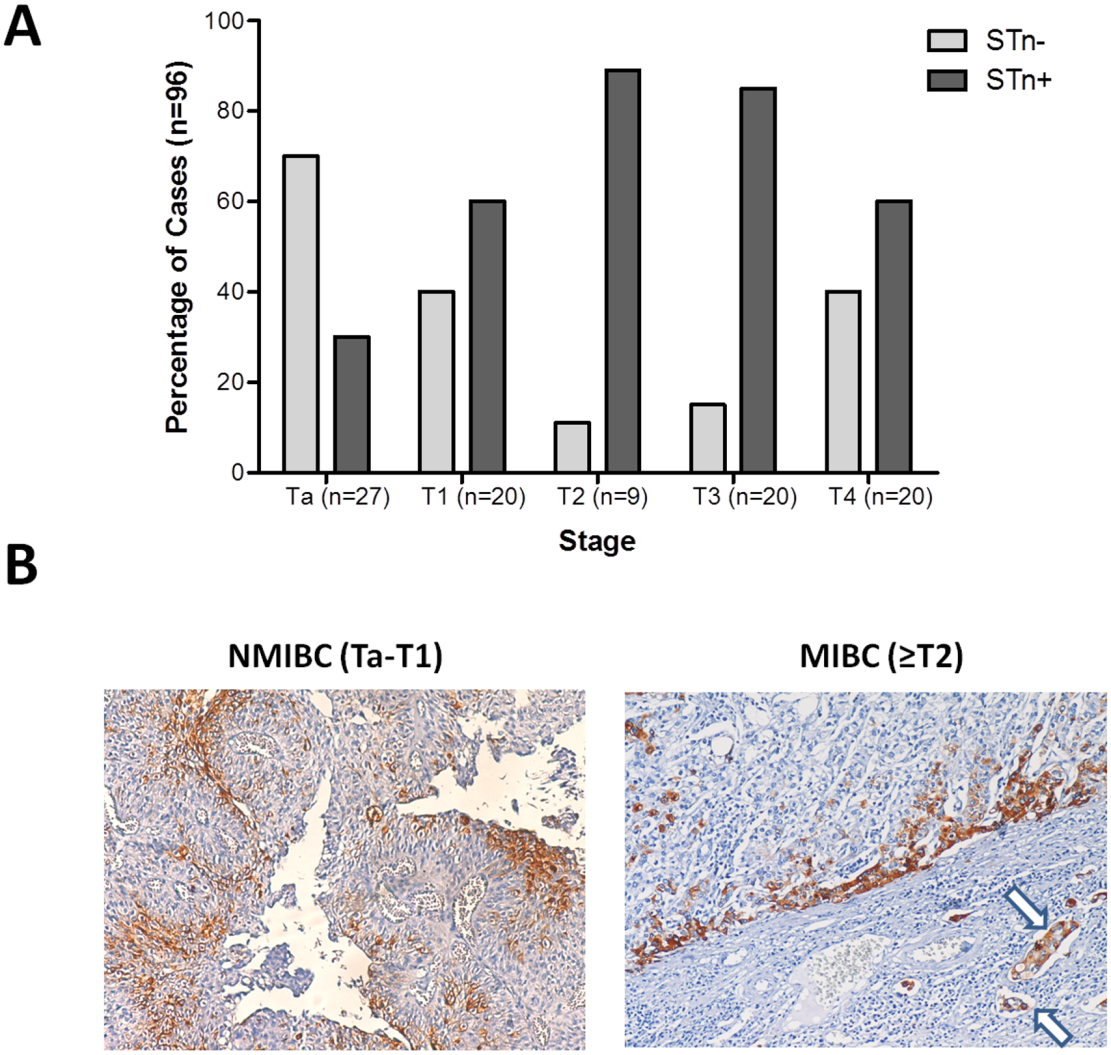
STn expression in different bladder tumors stages. (A) Distribution of STn negative and positive tumors along the different stages of bladder cancer; (B) Representative images of STn staining in NMIBC and MIBC. Left—NMIBC showing a predominance of STn positive cells in the superficial layers, away from the fibrovascular support; note vessels without positive cells. Right—MIBC showing the invasion front with STn positively stained cells; note positive STn urothelial cells in the vessels (arrow), suggesting possible involvement in metastasis.

The Tn antigen was observed in approximately 10% of NMBC and MIBC (Table 2) and its expression was residual, did not exceeding 5% of the tumour area and without any defined pattern. On the other hand, the STn antigen was detected in approximately 60% of the studied bladder tumours, which is in accordance with our previous findings [5]. The antigen was predominately expressed at the cell membrane, although cytoplasmic staining could also be observed. The STn antigen presented a focal expression that did not exceed 30% of the tumour area for the majority of the positive cases, irrespectively of their histological origin. STn was mainly expressed by dedifferentiated cells in tumours showing *lamina propria* (T1; 60%) and *muscularis propria* (≥ T2; approximately 60–90%) invasion; conversely the percentage of positive Ta was lower than 30% (*p*<0.001; Fig 3A). Although without statistical significance, in Ta tumours STn positive cells were mainly present in superficial tumour layers away from the vessels. Conversely, STn positive cells in T1 tumours (Fig 3B) were observed accompanying and/or invading the basal layer (Fig 3B), while in MIBC these cells were mostly found in the invasion fronts (Fig 3B) and invading and/or inside the vessels, which suggests a role in invasion and disease dissemination. Reinforcing these observations, the presence of STn antigen was statistically higher in MIBC when compared to NMIBC (*p* = 0.001, Table 3).

**Table 3:** Association between the evaluated markers and the stage of disease.

	Bladder Cancer		
	NMIBC	MIBC	P
	n (%)	n (%)	
**Tn**			
Negative	41 (87.2)	45 (91.8)	
Positive	6 (12.8)	4 (8.2)	0.461
**STn**			
Negative	27 (57.4)	12 (24.5)	
Positive	20 (42.6)	37 (75.5)	0.001
**pAKT**			
Negative	13 (28.9)	19 (38.8)	
Positive	32 (71.1)	30 (61.2)	0.312
**pmTor**			
Negative	30 (63.8)	33 (67.3)	
Positive	17 (36.2)	16 (32.7)	0.717
**pS6**			
Negative	22 (47.8)	28 (57.1)	
Positive	24 (52.2)	21 (42.9)	0.183
**PTEN**			
Negative	18 (38.3)	37 (82.2)	
Positive	29 (61.7)	8 (17.8)	<0.001

### PI3K/Akt/mTOR pathway in bladder cancer

The evaluation of the PI3K/Akt/mTOR/S6 pathway was done using antibodies for active phosphorylated forms of Akt (pAkt), mTOR (pmTOR), and S6 (pS6). PTEN, that negatively regulates Akt signalling, was also evaluated.

**Fig 4:**
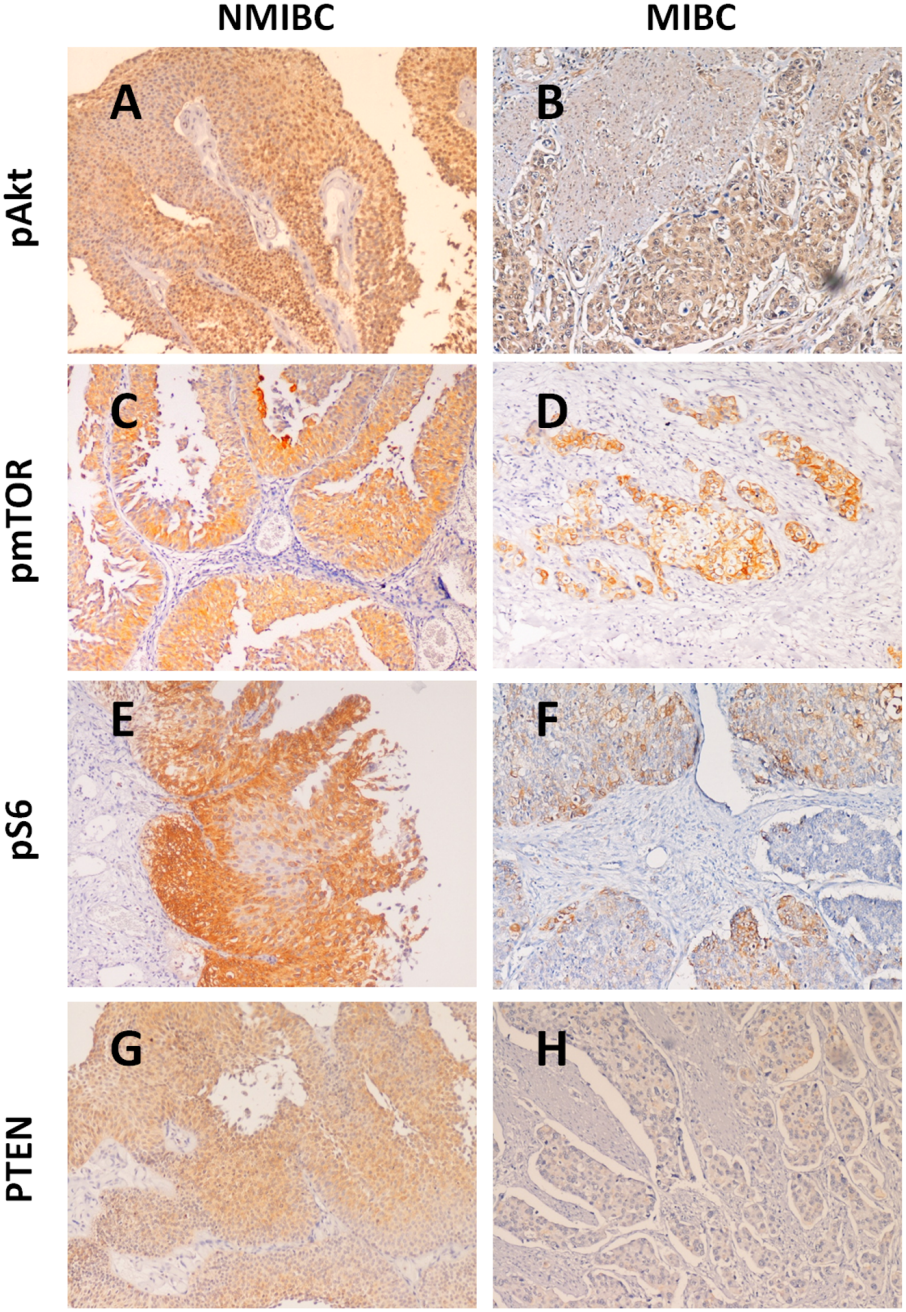
Expressions of pAkt, pmTOR, pS6 and PTEN in NMIBC and MIBC (40x magnification). A and B) pAKT nuclear and cytoplasmatic expression in NMIBC (A) and MIBC (B). In NMIBC cases pAkt presented a heterogeneous pattern with areas of different intensity of expression. In MIBC, stromal cells mainly in the areas close to the tumour showed higher expression. C and D) pmTOR cytoplasmic expression in NMIBC (C) and MIBC (D). In NMIBC pmTOR was expressed across several layers, although there was a more intense staining in the superficial ones. In MIBC positive cases pmTOR expression was focal. E and F) pS6 cytoplasmatic expression in NMIBC (E) and MIBC (F). In NMIBC pS6 expression was observed in all the superficial layers both in umbrella and differentiated cells. In MIBC the immunoreactivity was diffuse, however more present in basal and mitotic cells. pS6 expression was higher in the invasion front and in tumour infiltrating lymphocytes and endothelial cells. G and H) PTEN cytoplasmic and nuclear expressions in NMIBC (G) and MIBC (H). PTEN expression was higher in NMIBC compared to MIBC.

pAkt was detected both in the cytoplasm and nucleus. In NMIBC cases several areas with different intensity of expression were observed (Fig 4A), denoting a heterogeneous pattern that was not evident in MIBC (Fig 4B). Furthermore, stromal cells of MIBC positive cases showed enhanced staining intensity mainly in the areas close to the tumour. pmTOR immunoreactivity was cytoplasmic and, in occasional cases, nuclear. In urothelium with apparent normal histology pmTOR expression was restricted to superficial cell layers. In NMIBC pmTOR expression was evenly distributed across the several layers of urothelial cells, although there was a more intense staining in the superficial layers (Fig 4C). Moreover, several areas with variable staining intensity were observed, denoting a heterogeneous expression. In MIBC positives cases, pmTOR expression was focal and heterogeneous (Fig 4D). pS6 immunoreactivity was predominantly cytoplasmic. In NMIBC pS6 expression was noted in all the superficial layers, both in umbrella and differentiated cells (Fig 4E). The immunoreactivity of pS6 varied across the tumour cells. In MIBC pS6 presented a diffuse expression throughout the tumour, being more present in basal and mitotic cells (Fig 4F). Several positive cases presented increased pS6 staining intensity in the invasion front as well as pS6 expression in tumour infiltrating lymphocytes and endothelial cells.

Taking into account the extension of staining and its intensity, 62/94 (66%), 33/96 (34%) and 45/95 (47%) of the bladder tumours were considered positive for pAkt, pmTOR and pS6, respectively. A Spearman rho test showed that pAkt, pmTOR, pS6 expressions were significantly correlated (P<0.05) irrespectively of the tumour stage, thus in accordance with a fully active pathway. Furthermore, despite histological differences, these markers presented an equal distribution among the NMIBC and MIBC and could not be associated with muscle invasion (Table 3).

On the other hand, 37/92 (40%) of the tumours were considered positive for PTEN. PTEN was expressed in the cytoplasm and nucleus of the same cells, however with lower extension of expression in MIBC (33%, Fig 4G) compared to NMIBC (83%; Fig 4H). Moreover, the PTEN-negative phenotype was significantly associated with muscle invasion (Ta and T1; p<0.001, Table 3), which may contribute to maintain an active PI3K/Akt/mTOR/S6 pathway in these cases.

### Tn, STn, PI3K/Akt/mTOR pathway and Cancer-specific Survival

**Fig 5:**
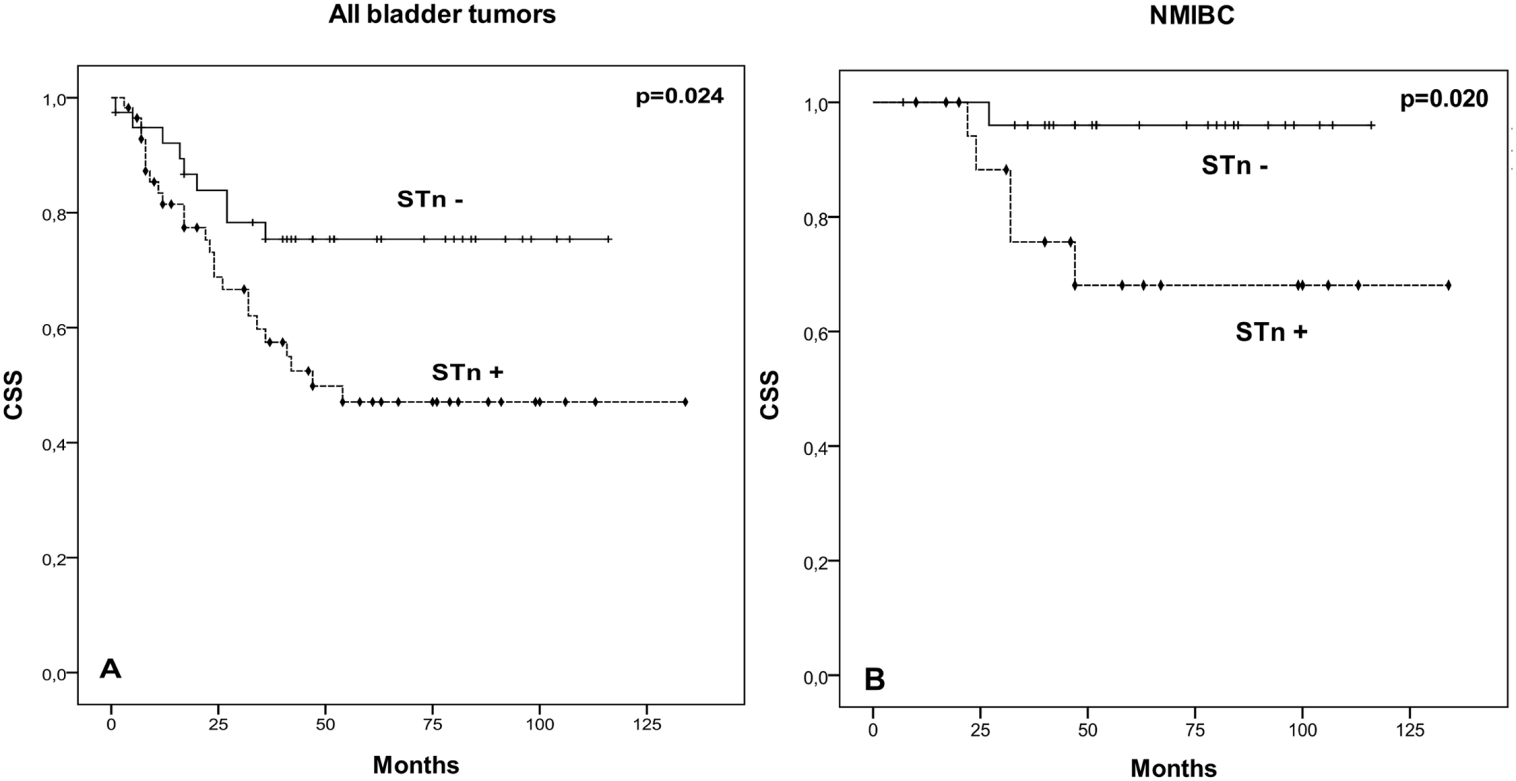
Effect of STn expression in cancer-specific survival (CSS). Kaplan–Meier analysis showing the association between STn and CSS in: (A) all studied bladder cancer patients; (B) NMIBC patients. Comparison performed by log-rank test (A: p = 0.024; B: p = 0.020); + censored STn negative tumours; ⧫ censored STn positive tumours.

**Fig 6:**
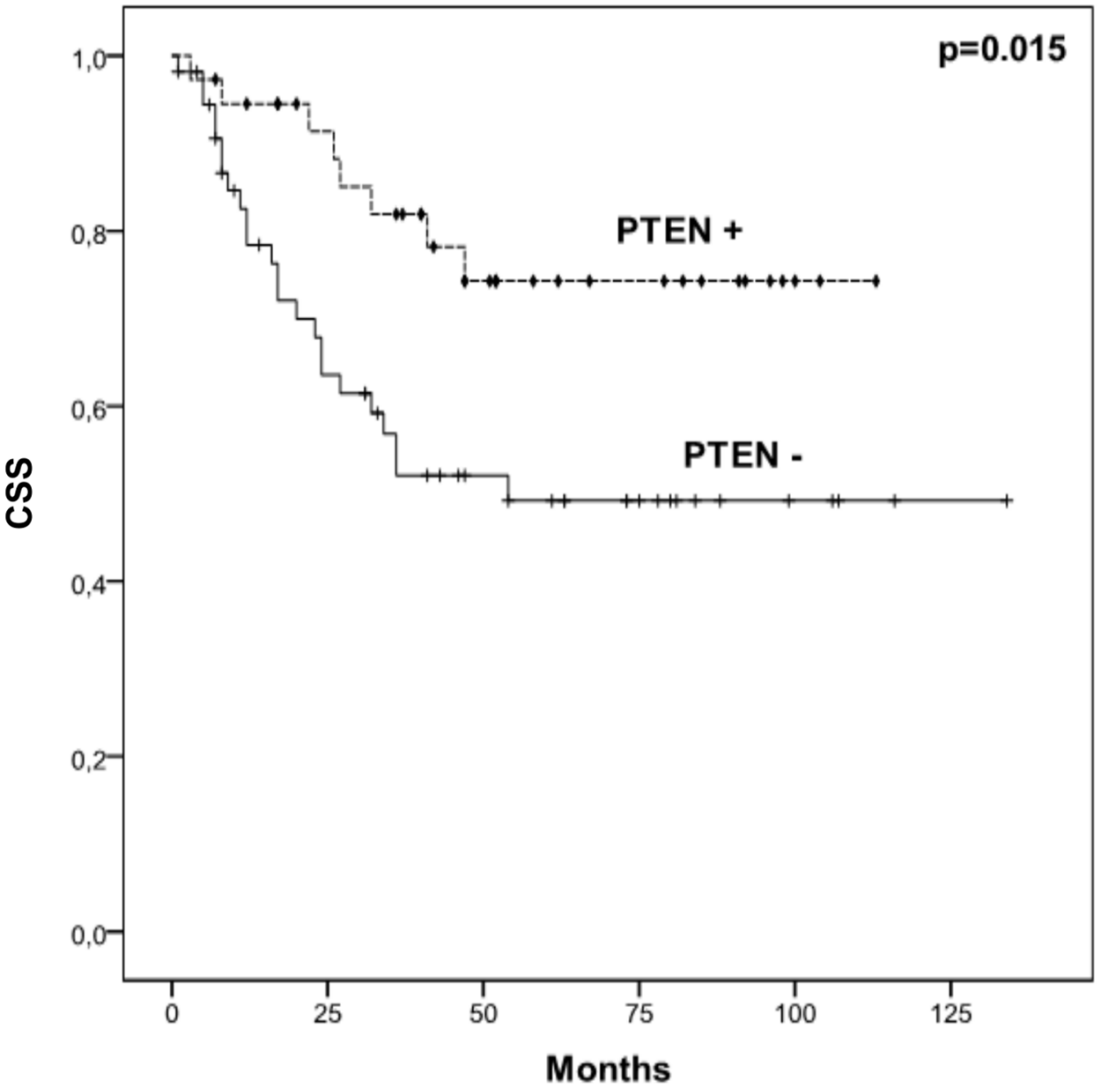
Effect of PTEN expression and cancer-specific survival (CSS) in the studied patients. Kaplan-Meier analysis showing the effect in CSS of PTEN expression in all studied bladder cancer patients. Comparison performed by log-rank test (p = 0.013); + censored PTEN negative tumours; ⧫ censored PTEN positive tumours.

A Kaplan-Meier analysis was used to evaluate associations between the addressed biomarkers and the cancer-specific survival of patients. We observed that patients bearing STn expressing tumours had a lower CSS, irrespectively of their stage (p = 0.024; Fig 5A). This was also observed when evaluating NMIBC alone (p = 0.020; Fig 5B). More importantly, among NMIBC, STn expressing T1 tumours presented lower CSS than negative tumours (p <0.05). Moreover, multivariate Cox regression analysis adjusted to potential confounders, namely age, gender, stage, grade, recurrence status, presence of concomitant CIS was performed. We found that STn is an independent prognostic marker of worst CSS (HR = 11.836; 95%CI: [1.063–131.7]; p = 0.044). Contrasting with STn, positive Tn, pAkt, pmTOR and pS6 tumours showed no differences in CSS compared to negative lesions, irrespectively of their stage. We have also observed that patients harbouring PTEN-negative tumours had lower CSS (p = 0.015, Fig 6). More studies are necessary to determine if the lack of suppressive effect of PTEN over PI3K/Akt/mTOR may account for these findings.

**Fig 7:**
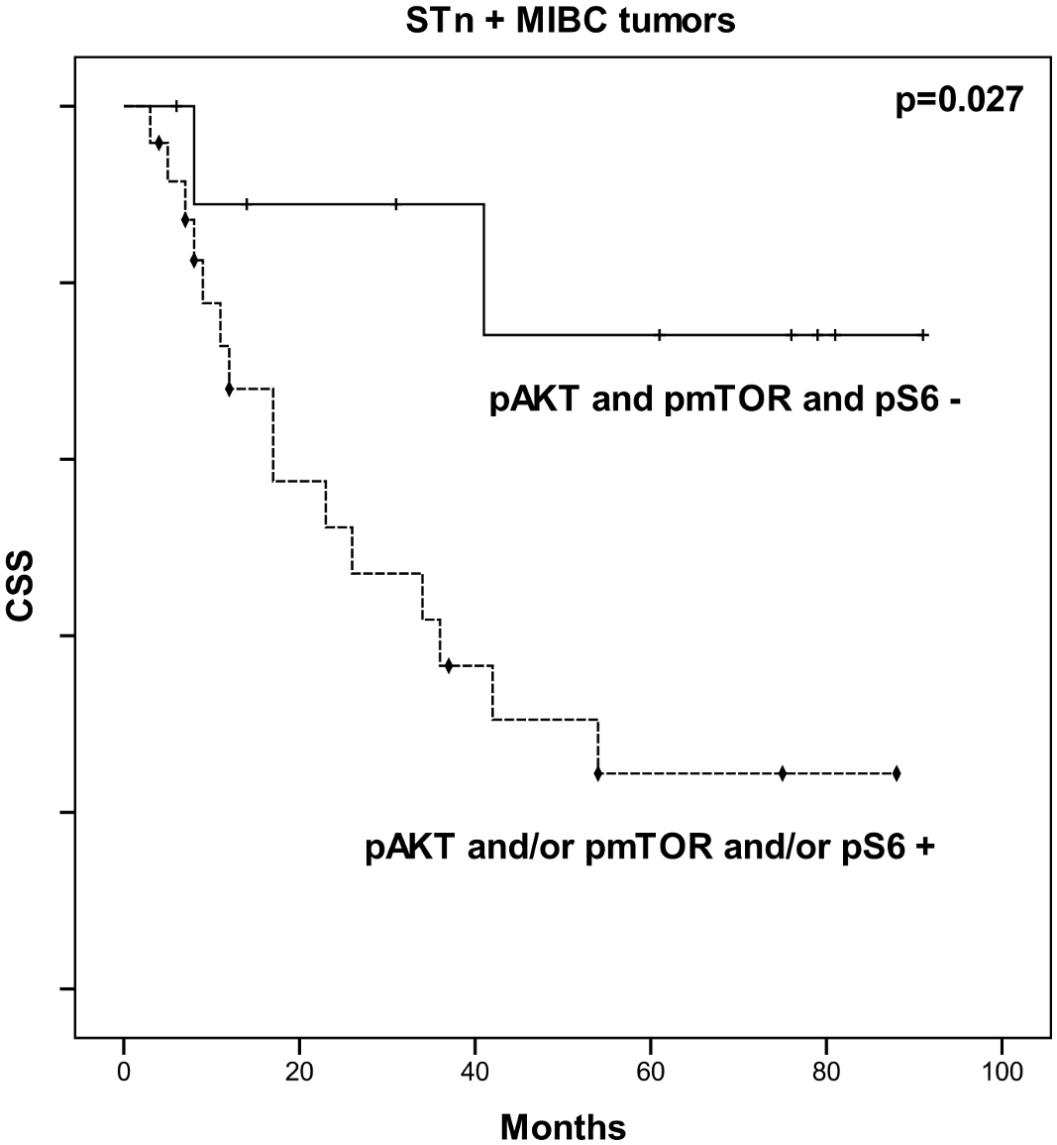
Effect of PI3K/Akt/mTOR pathway activation in cancer-specific survival (CSS) of patients with STn positive MIBC. Kaplan–Meier analysis showing the association between pAKT, pmTOR and pS6 expressions in the CSS of STn positive tumors MIBC: Comparison performed by log-rank test (p = 0.027); + censored pAKT and pmTOR and pS6 negative tumours; ⧫ censored pAKT and/or pmTOR and/or pS6 positive tumours.

Based on these observations and aiming to improve the prognostic value of STn in the context of late stage disease (MIBC), we have comprehensively integrated the information from STn and PI3K/Akt/mTOR pathway biomarkers. According to Fig 7, the introduction of PI3K/Akt/mTOR pathway molecules allowed discriminating STn positive MIBC tumours with worst CSS (p = 0.027). Furthermore, multivariate Cox regression analysis (adjusted to age, stage, recurrence status, presence of concomitant CIS and metastasis) revealed that the presence of PI3K/Akt/mTOR pathway molecules in STn+ MIBC is independently associated with approximately 6-fold risk of death by cancer (HR = 5.662; 95%CI: [1.093–29.323]; p = 0.039). These observations suggest, for the first time, that the combination of STn and mTOR pathway biomarkers may hold potential to improve the stratification of advanced stage bladder tumours; however corroboration in larger series is mandatory.

### Inhibition of the PI3K/Akt/mTOR pathway in animal models

**Fig 8:**
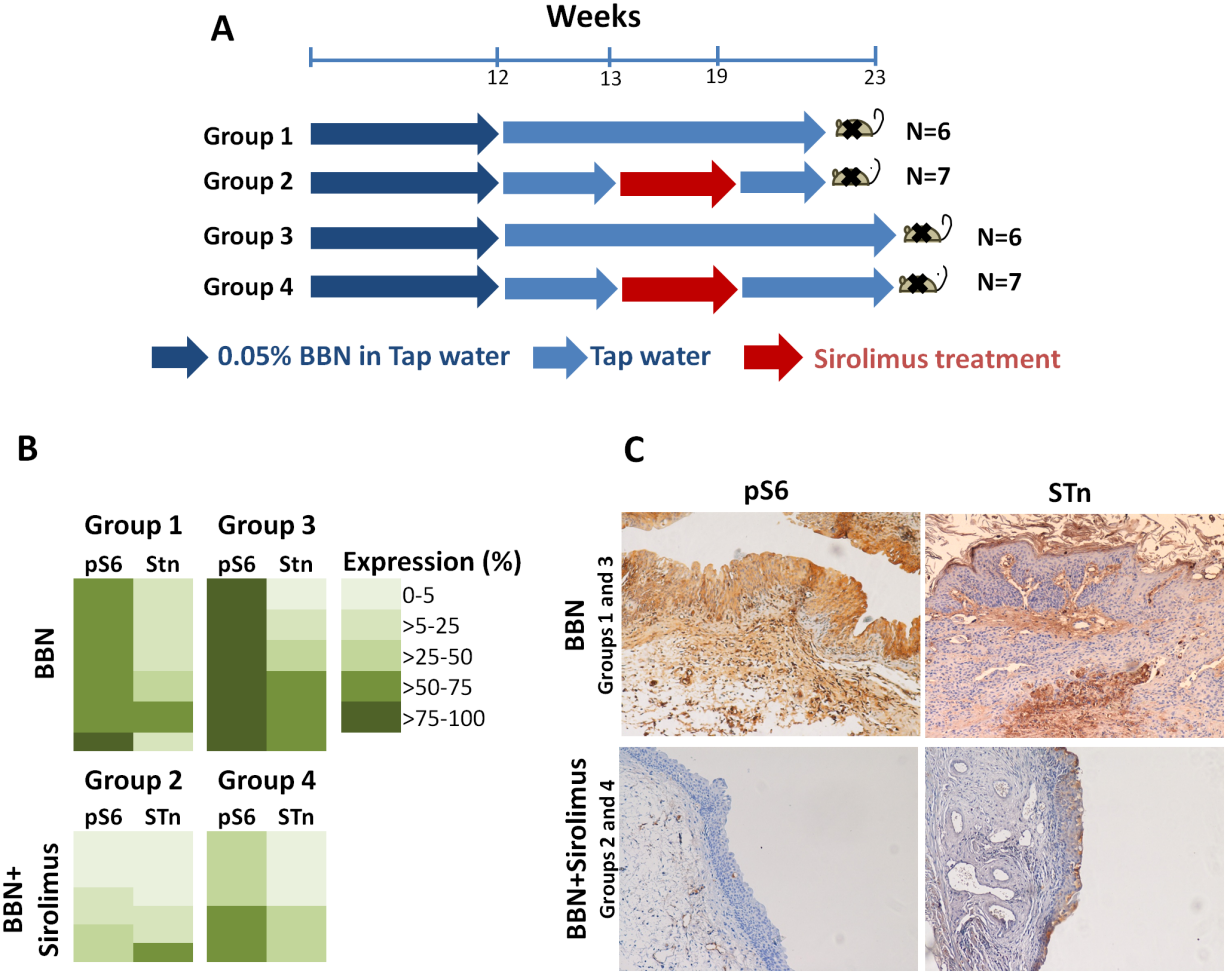
STn and pS6 expressions in bladder tumours from BBN-exposed male ICR mice with or without the administration of mTOR-inhibitor sirolimus (rapamycin). A) Experimental design to determine the sirolimus effect on STn and pS6 expressions in a model of urothelial carcinogenesis (male ICR mice). B) Expression of STn and pS6 in BBN-derived urothelial tumours in the presence and absence of sirolimus. BBN-induced bladder tumours (Groups 1 and 3) overexpressed STn and pS6, which was more pronounced in Group 3, after longer lifespan. Exposure to sirolimus decreased the number of invasive lesions in groups 2 and 4 (data not shown) and, concomitantly, decreased the expressions of STn and pS6. C) Histological sections showing the expressions of STn and pS6 in BBN-induced urothelial tumours before and after treatment.

BBN-induced mice bladder tumours mimicking the histology and molecular nature of human cancers [20,21], were screened for STn and pS6, the downstream effector of mTOR pathway. We observed no STn expression in the healthy mice urothelium, in accordance with previous observation for the healthy human bladder [5]. In mice healthy urothelium pS6 expression was below 20%, thus underexpressed when compared with BBN-exposed mice (Fig 8). In the control groups (Group 1 and 3, Fig 8A), the exposure to BBN led to the development of invasive tumours in 70–90% of the studied mice. Concomitantly, 83–100% of the invasive lesions overexpressed the STn antigen and all significantly overexpressed pS6 (Fig 8B). This demonstrated that BBN-induced lesions were able to recapitulate the association between altered glycosylation and an activated PI3K/Akt/mTOR pathway previously observed in advanced stage human tumours. The STn antigen was mainly found in cells adjacent to the basal layer and in those invading the stroma, as previously observed in human tumours (Fig 8B and 8C). Conversely, pS6 presented a more diffuse expression, again in accordance with the pattern observed in human lesions (Fig 8B and 8C). A comparison between groups 1 and 3 further highlighted that extended lifespan did not alter the number of invasive lesions, but significantly increased STn and pS6 overall expressions in each tumour (p<0.05; Fig 8B), highlighting the more aggressive nature of Group 3 lesions.

In the sirolimus-treated groups (Groups 2 and 4; Fig 8A) a smaller number of mice developed invasive tumors (20–40%) when compared to the controls (Groups 1 and 3). Moreover, only 43% of the mice treated with sirolimus overexpressed the STn antigen, irrespectively of the experience periods. Still, the extension of STn expression was significantly decreased in STn-positive tumours when compared to the control groups (Fig 8B and 8C). Following the same tendency, the pS6 protein was only overexpressed in 29% of the cases in Group 2 and the extension of expression was also significantly decreased (Fig 8B and 8C). Contrastingly, the expression of pS6 in Group 4 was higher than in Group 2, again translating the higher aggressive nature of tumours obtained after longer lifespan. Despite these observations, sirolimus treatment promoted a significant reduction in the percentage of positive pS6 cells in Group 4 mice when compared to Group 3 (p<0.05; Fig 8B and 8C). Altogether, sirolimus administration effectively reduced tumour burden and promoted a significant reduction in the expression of STn and pS6 markers.

## Discussion

Due to their high molecular heterogeneity, advanced stage bladder tumours present a significant prognostication and treatment hurdle. In this context, much controversy exists regarding the potential of conventional cancer biomarkers, urging the identification of novel molecules capable of aiding disease personalization. Furthermore, advanced stage bladder cancer remains an orphan disease in terms of therapeutics, as the only available options continue to be surgery and conventional chemotherapy [22]. The introduction of targeted therapeutics is therefore warranted.

In a previous explorative study we have observed that altered protein glycosylation translated by STn overexpression was a salient feature of a subset of advanced stage tumours [5]. Herein we have started by investigating the expression of STn precursor, the Tn antigen, in bladder tumours. We observed that this antigen presented a very low expression in bladder tumours and was not associated with any particular stage of the disease. These findings suggest that the Tn antigen is rapidly sialylated or capped with more extended glycans in bladder tumours. Moreover, we have confirmed that STn expression is more associated with muscle invasive than non-muscle invasive disease in a larger patient set, suggesting that sialylation plays a key role in stopping protein glycosylation in advanced stage bladder tumours. Furthermore, we have provided new insights regarding its correlation with decreased survival, as previously observed for digestive track tumours [23–25]. Accordingly, we and other authors have shown that STn expression is responsible by the modulation of cell surface glycoprotein functions in ways that favour malignant phenotypes in gastric [26], breast [27] and bladder [5] cancers. Namely, STn expression altered the adhesive properties of cancer cells, possibly by impairing integrin function [26,27]. Furthermore, it enhanced cell motility, invasion [26,27] and epithelial-to-mesenchymal transition, a key event leading to metastasis [28]. We have also demonstrated that STn expression protects bladder cancer cells from adverse host immune responses [6]. Namely, it impaired dendritic cell maturation inducing a tolerogenic phenotype and limiting their capacity to trigger protective anti-tumour T-cell responses [6]. In resume, a significant amount of data supports a key role of STn in disease progression and dissemination, making of STn antigen, and in particular STn-glycoproteins, potential anti-cancer targets. Nevertheless, there is scarce information about the molecular nature of this subset of STn-expressing aggressive tumours and consequently about the best therapeutic options.

Foreseeing a more accurate patient stratification we have also addressed the expression of PI3K/Akt/mTOR pathway markers in bladder tumours. In our series the activation of mTOR pathway proteins did not discriminate the stage of disease. Moreover it did not allow, by itself, the identification of patients facing worst prognosis, which is in accordance with recent publications [29,30]. However, we found that PTEN expression, which exerts a suppressive effect over the PI3K/Akt/mTOR pathway, was decreased in advanced stage tumours, in accordance with previous observations [31–34]. Furthermore, PTEN-negative MIBC presented worst cancer-specific survival in comparison to PTEN-positive lesions. More studies are needed to determine if the lack of suppressive effect over the PI3K/Akt/mTOR may account for poorer outcome. Interestingly, we have also observed that the overexpression of PI3K/Akt/mTOR pathway biomarkers decisively associated with worst CSS in STn positive advanced stage tumours, which currently lack effective therapeutics. These findings lead us to hypothesize that this subset of more aggressive bladder tumours may benefit from multi-targeted approaches combining mTOR-inhibitors and guided therapeutics against STn-expressing cells. However these are preliminary insights from a relatively low number of patients. More studies involving a large population are warranted to confirm these observations. It will also be important to evaluate other outcomes of aggressiveness, namely response to conventional therapeutics and metastasis development.

Our study also reinforced that bladder tumours present extensive activation of the PI3K/Akt/mTOR pathway irrespectively of their histological nature, as described in previous publications [32,35]. Such findings contribute to support the idea that most bladder tumours may be good candidates for mTOR-inhibitors therapeutics. Accordingly, mTOR-inhibitors have been extensively explored in pre-clinical settings and two phase I/II clinical trials for bladder cancer are ongoing [36]. In particular our group has demonstrated that the combination of everolimus with cisplatin or gemcitabine decreased the proliferation of bladder cancer cell lines in comparison to the chemotherapy agent alone [14,37]. More recently we conducted studies in mice bearing chemically-induced tumours mimicking the histological and molecular nature of human tumours [20]. We concluded that administration of mTOR-pathway inhibitor sirolimus (rapamycin) effectively reduced the frequency of invasive lesions. Using the same animal model, we have now confirmed the anti-cancer activity of sirolimus in the context of aggressive bladder disease. Namely, we observed a significant reduction in tumour burden accompanied by a loss of pS6 expression, thus in accordance with the expected mechanism of action of the drug. Moreover, we are describing for the first time that chemically-induced bladder tumours expressed the STn antigen, thereby mimicking the glycosylation pattern of human cancers. These observations are of particular importance due the lack of accurate models to access the biological role of this antigen. In fact most established cancer cell lines express residual amounts of this antigen, denoting a dependence on the tumours microenvironment. We believe that BBN-induced tumours may now constitute key models to develop successful therapeutics against STn positive bladder lesions. Moreover importantly, we have concluded that the administration of sirolimus contributed to reduce the number of STn positive cells. These observations reinforce a possible association between STn and an active PI3K/Akt/mTOR pathway in invasive tumours, as suggested upon the evaluation of human cancers. It also points out that sirolimus may constitute a valuable approach to manage STn and PI3K/Akt/mTOR-positive, which face worst OS. Still, these preliminary evidences and more in depth studies are needed before progressing to clinical phases. Namely, it will be important to support these findings in other models such as patient-derived xenografts and compare the effect of sirolimus with conventional chemotherapeutics for bladder cancer (cisplatin/gemcitabine-based regimens).

In resume, we have demonstrated that the STn antigen is a biomarker of poor prognosis, particularly in MIBC. We also suggest the existence of potentially more aggressive subgroup of STn positive MIBC characterized by an active mTOR-pathway. Such observations also provide the first link between these two apparently unrelated events in bladder cancer (altered glycosylation and the PI3K/Akt/mTOR-pathway activation). Using animal models we have also concluded that the administration of mTOR-pathway inhibitor sirolimus offers potential against these highly malignant tumours. More validation studies are now warranted to set the pace for clinical trials. Taking into consideration its cell-surface nature and key role played by STn malignancy, specific antibody-based therapeutics can also be envisaged [22,38]. The combination of these approaches may provide novel ways to improve MIBC management, which remains an orphan disease in terms of innovative treatments [22].
